# Leber’s Hereditary Optic Neuropathy (LHON): Clinical Experience and Outcomes after Long-Term Idebenone Treatment

**DOI:** 10.3390/life13102000

**Published:** 2023-09-30

**Authors:** George Baltă, Georgiana Cristache, Andreea Diana Barac, Nicoleta Anton, Ileana Ramona Barac

**Affiliations:** 1 Doctoral School, University of Medicine and Pharmacy “Carol Davila” Bucharest, 050474 Bucharest, Romania; george.balta@drd.umfcd.ro; 2 Faculty of Medicine, University of Medicine and Pharmacy “Carol Davila” Bucharest, 050474 Bucharest, Romania; andreeadiana.e.barac@stud.umfcd.ro; 3 Department of Ophthalmology, University of Medicine and Pharmacy “Grigore T. Popa” Iasi, 700115 Iasi, Romania; nicolofta@gmail.com; 4 Department of Ophthalmology, University of Medicine and Pharmacy “Carol Davila” Bucharest, 050474 Bucharest, Romania; ramona.barac@umfcd.ro

**Keywords:** Leber’s hereditary optic neuropathy, Idebenone, long-term regimen

## Abstract

Background: Leber’s hereditary optic neuropathy (LHON) is a rare disease. Large studies are difficult to conduct; therefore, case reports provide valuable data. Since 2015, patients have been treated with Idebenone. The aim of this paper is to share our experience with diagnosing and managing patients in different stages of LHON. Methods: We designed a case series study, including four patients undergoing genetic testing and ophthalmologic examination. Criteria for Idebenone administration and follow-up were presented. Results: All patients had mutation 11778G>A in MT-ND4. The first patient, an 82-year-old man, with long history of vision loss, had no indication for Idebenone. Two additional cases emerged within the same family: a 40-year-old brother and a 31-year-old sister. Both received Idebenone, with good outcomes only for the female. After a one-year regimen, they were lost to follow-up. The fourth patient, a 46-year-old man, was diagnosed in the subacute stage. Idebenone administration was deferred, allowing progression of visual field defects. After 17 months of treatment, visual improvement appeared. The treatment was continued for 36 months, with short interruptions, resulting in good outcomes. Conclusions: Our study demonstrated positive results with long-term Idebenone use. Contrary to medical literature, our female patient had a favorable evolution, despite the delayed diagnosis.

## 1. Introduction

Leber’s hereditary optic neuropathy (LHON) is the most frequent mitochondrial disease, maternally transmitted, with a prevalence of 1 in 45,000 in the European population, affecting predominantly young adult males [1]. It belongs to the category of rare diseases, which are defined by the European Union as conditions affecting fewer than 1 in 2000 people in the general population [2].

The disease was first reported in 1871 by Theodor Leber, a German ophthalmologist who presented the main symptoms observed [3]. Only in 1988, Wallace managed to explain the implication of mitochondrial DNA mutations in pathogenesis [4].

Individuals diagnosed with LHON may experience painless, gradual loss of central vision and altered color perception. Approximately 75% of cases involve a sequential loss of vision in both eyes, whereas the remaining 25% experience simultaneous vision loss [5,6]. The symptoms arise due to selective degeneration and loss of retinal ganglion cells (RGCs) and their axons, with the papillo-macular fibers being impacted early on, followed by the entire optic nerve [7]. These events are a consequence of point mutations in mitochondrial DNA. There are three main genetic mutations, accounting for nearly 90% of all cases of LHON (m.3460G>A in MT-ND1, m.11778G>A in MT-ND4, and m.14484T>C in MT-ND6). They affect the synthesis of proteins involved in the complex I of the mitochondrial respiratory chain, resulting in the accumulation of reactive oxygen species (ROS) and damage of RGCs [8]. It is worth noting that m.11778G>A is the most commonly observed mutation among the affected population worldwide, occurring in 60–80% of cases [9]. Interestingly, the pathogenesis of LHON impacts RGCs and their axons, yet the reason for this unique vulnerability remains unclear [10]. There are scientific theories sustaining that RGCs possess unmyelinated axons as a protective measure against light radiation that may harm the retinal pigment epithelium, which results in the absence of saltatory action potential. This requires a significant amount of energy, which is met by mitochondrial aggregation. Furthermore, RGCs are highly sensitive to energy depletion and ROS [11,12].

It is noteworthy that not all individuals carrying the mutation will exhibit symptoms, as the penetrance of mutations varies. Research indicates that approximately 50% of males and 10% of females with different mutations will eventually develop the disease [5,13].

While recent gene therapy research has shown promising results, further extensive clinical trials are necessary to develop new agents that can improve the prognosis of the disease and the quality of life [8,14].

In 2015, the European Medicines Agency (EMA) approved Idebenone (Raxone^®^, Santhera Pharmaceuticals GmbH, Lörrach, Germany) for treating patients with Leber’s Hereditary Optic Neuropathy (LHON) at 900 mg/day in three doses. Idebenone is a synthetic hydrosoluble analogue of Co-enzyme Q10 that works by transferring electrons directly to complex III of the mitochondrial respiratory chain, bypassing the dysfunctional complex I [15]. Since its approval, Idebenone has become the primary antioxidant medication used to treat LHON and has been thoroughly researched. Studies have shown that Idebenone can increase ATP production and decrease ROS levels in cells from LHON patients that come into contact with it [16].

However, the RHODOS study sparked controversy when some patients responded to therapy while others did not [17]. Subsequent research discovered that patients who experienced improved visual acuity while taking Idebenone continued to see improvements even after stopping treatment [18].

In order to establish guidelines for diagnosis, treatment, and follow-up, a panel of experts from Europe and North America published an international consensus on the staging and therapeutic management of LHON [19].

According to the latest research, LHON has four clinical stages: asymptomatic (mutation carriers), subacute (<6 months from onset), dynamic (6–12 months), and chronic (>12 months) [19]. Based on this classification, Idebenone should be started as soon as possible in patients with a disease history of less than 1 year. In patients with chronic disease, the treatment is not recommended [19]. The duration of treatment may vary depending on the patient’s response. When treatment is effective, it should continue for one year after the plateau of improvement. Recent studies [15,20] recommend administering Idebenone for more than 24 months, as vision can deteriorate before improving despite treatment initiation, followed by subsequent improvement. An extended duration of Idebenone administration can increase the chances of clinically relevant recovery.

The aim of this paper is to present our experience with the diagnoses and management of four LHON patients. The study is designed as a case-based analysis and describes the evolution of three patients with longstanding (chronic stage) disease and one patient diagnosed in the subacute stage. Three of these patients were treated with Idebenone, and the results were studied.

## 2. Materials and Methods

We conducted an observational, descriptive case series study on patients with LHON, focusing on diagnosis, management and outcomes with and without treatment (Idebenone).

Between 2018 and 2023, our center diagnosed four patients with LHON. Prior to 2017, there was no specific treatment available for LHON patients in Romania. Three of our patients were eligible for Idebenone administration through the National Health Program for Rare Diseases, as they met the criteria for treatment initiation. To receive treatment, patients needed to meet at least two criteria; one of which was mandatory (positive genetic testing for LHON) and one optional criterion related to LHON-specific signs and symptoms developed no more than five years prior to treatment start. Optional criteria included painless, acute/subacute vision loss, central or ceco-central visual field scotoma, decreased visual acuity below 1.0 logMAR within 12 months of clinical onset, dyschromatopsia, lack of response to corticosteroids administered for 15–30 days, optic disc pseudo-edema, and alterations in RGCs and their axons.

We conducted genetic testing on all patients by obtaining mitochondrial DNA from ethylenediaminetetraacetic acid (ETDA) venous blood samples. Polymerase chain reaction (PCR) was used to analyze the relevant areas of MT-ND1, MT-ND4, and MT-ND6 mitochondrial genes, followed by Sanger sequencing. The obtained sequences were then compared with reference sequences (ENST00000361390–MT-ND1, ENST00000361381–MT-ND4, and ENST00000361681–MT-ND6).

Before undergoing genetic diagnosis, the patients underwent an extensive ophthalmologic examination that included various tests, such as best-corrected visual acuity (BCVA), using ETDRS chart, slit lamp exam (anterior and posterior segment examination), intraocular pressure, fundus photography using 3D Topcon DRI OCT Triton Swept Source, OCT RNFL using 3D Topcon DRI OCT Triton Swept Source and Spectralis–OCT, static perimetry (Humphrey Field Analyzer, using 24-2 and 10-2 SITA standard protocol), and Goldmann kinetic perimetry—used for patients with important decreased vision.

For patients who met the treatment criteria, we tried to start the therapy as soon as possible. Idebenone was administered orally, 900 mg/day, and divided in 3 doses.

To evaluate the efficacy of Idebenone treatment, we assessed the patients’ visual acuity, visual fields, and OCT RNFL. Follow-up visits were scheduled approximately every 3 months for the first 6 months and then every 6 months. Although our patients were unable to follow the exact appointments, we tried to approximate the periods.

The criteria proposed by the Romanian National Health System were used to evaluate the efficacy of Idebenone treatment for LHON patients. Clinically relevant recovery (CRR) was defined as improvement of at least 10 ETDRS letters (2 lines on ETDRS chart or 0.2 logMAR units) for patients with visual acuity 1.0 logMAR or less or improvement from off-chart (>1.69 logMAR) to on-chart by at least 5 ETDRS letters—one full line (≤1.60 logMAR). Clinically relevant stabilization (CRS) was important for early diagnosed patients, with visual acuity less than 1.0 logMAR at baseline, who maintained the same visual acuity at the last visit.

Idebenone administration in Romania cannot exceed 36 months, as long as the patient shows relevant visual improvement. However, if the patient shows no improvement, the treatment should be ceased after 24 months.

## 3. Results

Four individuals, consisting of three males and one female, received a diagnosis of LHON after undergoing genetic testing due to clinical suspicion. The genetic testing confirmed a positive mutation of 11778G>A in MT-ND4.

**Figure 1:**
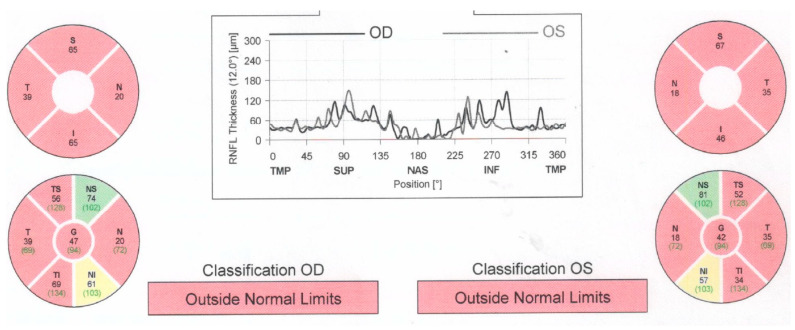
Optical coherence tomography (OCT) report showing significant retinal nerve fiber layer thinning in both eyes, with normal thickness preserved in supero-nasal sector. The thickness is also displayed in color-coded graphs: red—outside of normal limits, yellow—borderline, green—within normal limits. (OD—right eye; OS—left eye; RNFL—retinal nerve fiber layer; S, SUP—superior; T, TEMP—temporal; N, NAS—nasal; I, INF—inferior; TS—supero-temporal; NS—supero-nasal; TI—infero-temporal; NI—infero-nasal).

Case number one was an 82-year-old man who had experienced vision loss 60 years prior and was initially misdiagnosed with non-arteritic ischemic optic neuropathy and retrobulbar optic neuritis. Both eyes had a visual acuity of 1.8 logMAR (counting fingers at 1 m), with an intraocular pressure of 13 mmHg in the right eye and 14 mmHg in the left eye. Bilateral pseudophakia (posterior chamber intraocular lens) was observed during anterior segment slit lamp examination, and fundus examination revealed bilateral optic disc atrophy, which was confirmed by optical coherence tomography (OCT) (Figure 1).

Goldmann kinetic perimetry was performed, showing bilateral central scotomas and importantly constricted visual fields. Kinetic perimetry was utilized due to the patient’s decreased visual acuity, which made Humphrey perimetry challenging to perform.

Given the extensive period between symptom onset and diagnosis, case number one was not deemed suitable for Idebenone treatment.

**Figure 2:**
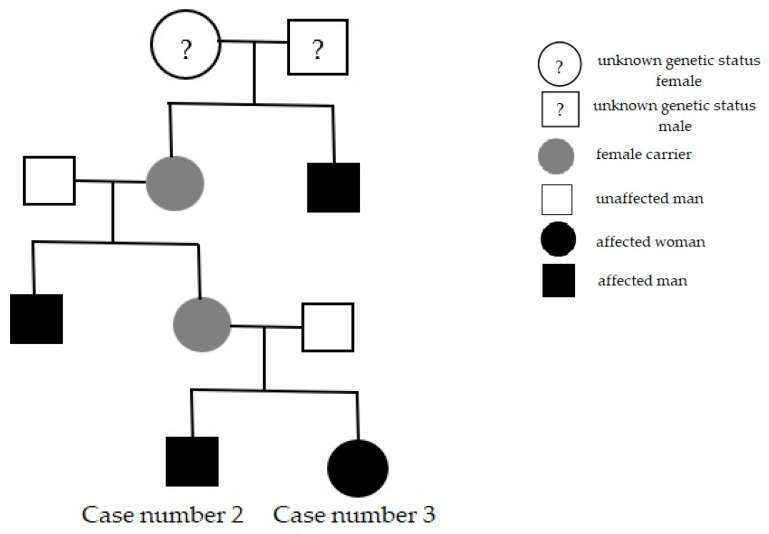
Family pedigree presentation, in which the last subjects are our patients (Cases number two and three).

Cases number two and three were found in the same family: the 40-year-old brother and the 31-year-old sister, who also reported a strong family history; their mother’s brother and their grandmother’s brother had vision loss early in life (Figure 2).

**Figure 3:**
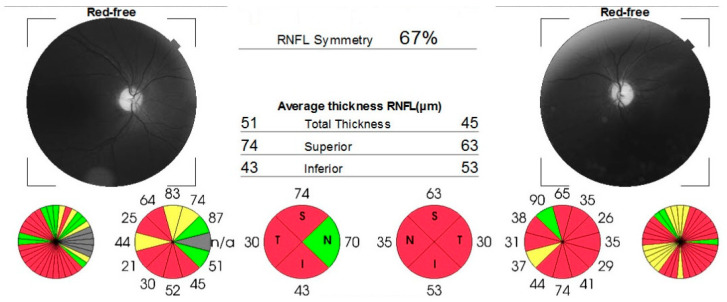
OCT report, showing significant thinning of retinal nerve fibers layer in both eyes. Areas with low signal quality are displayed in gray.

Case number two (the brother) experienced subacute, painless bilateral visual loss (occurring simultaneously and gradually over approximately one week), with no significant further progression, starting 5 years before presentation in our center. In the past, he was diagnosed with bilateral optic disc atrophy of an unknown cause. The patient had a history of smoking for 10 pack years. Visual acuity was 1.60 logMAR in the right eye and 2 logMAR in the left eye, and intraocular pressure was 19 mmHg and 17 mmHg, respectively. The anterior segment aspect was normal for his age. During the dilated fundus exam, diffuse optic disc pallor was observed in both eyes, with larger pallor than the cup. An OCT scan (Figure 3) confirmed optic nerve atrophy, indicating a loss of nerve fibers in the superior, inferior, and temporal quadrants of the right eye, as well as in all four quadrants of the left eye. Due to decreased visual acuity, the patient underwent Goldmann kinetic perimetry, which revealed a central scotoma and temporal quadrantanopia in the right eye and a central scotoma in the left eye.

**Figure 4:**
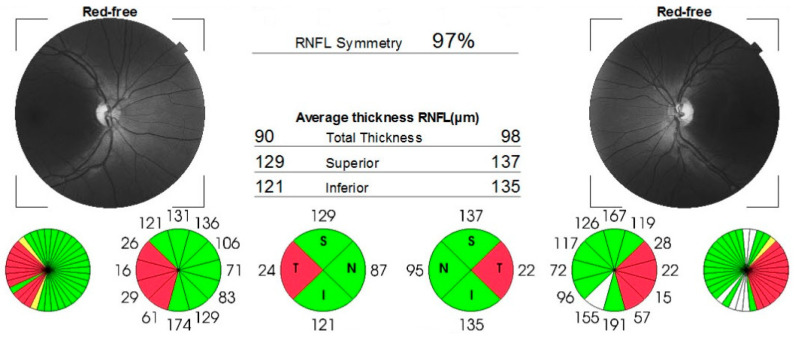
OCT report confirming thinning of nerve fibers layer in temporal sector. White color indicates thicker RNFL.

Case number three (the sister) complained of acute, painless vision loss in her right eye, noticed 2 years before presentation. She was earlier misdiagnosed with steroid-resistant optic neuritis and normotensive glaucoma. Visual acuity was 0.5 logMAR in the right eye and 0.1 logMAR in the left eye. The intraocular pressure was 17 mmHg in right eye and 18 mmHg in left eye. Both eyes showed no unusual findings during the anterior segment examination. However, the fundus examination revealed asymmetric optic disc pallor, particularly in the temporal sector of both eyes. This was confirmed by the loss of temporal retinal nerve fibers on the OCT scan (Figure 4) and ceco-central scotomas identified during the 24-2 Humphrey visual field test.

Both patients were prescribed Idebenone according to treatment criteria. Patient two was recommended to quit smoking, but, unfortunately, was unable to comply.

During the first follow-up after 3 months, different outcomes were observed.

Patient number two did not experience any significant improvement in their clinical or psychophysical condition, with their visual acuity and fields remaining stable.

**Figure 5:**
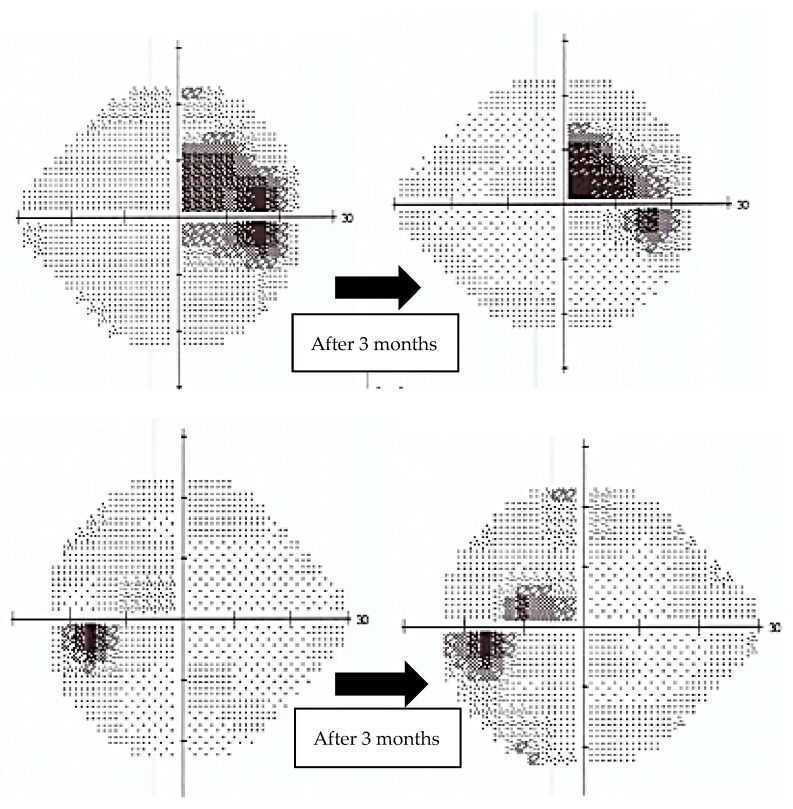
Evolution of 24-2 Humphrey Visual Field (VF) from the beginning of treatment to 3 months follow-up for case number three. The plots show significant improvement. (**top**: right eye, **bottom**: left eye).

In contrast, case number three exhibited a remarkable increase in visual acuity. (0.2 logMAR vs. 0.5 logMAR at baseline in right eye and 0 logMAR vs. 0.1 logMAR at baseline in left eye), with a positive trend in mean deviation (MD) on 24-2 Humphrey VF (right eye: MD −6.65 dB, *p* < 0.5%—at diagnosis vs. −4.13 dB, *p* < 0.5%—after 3 months of Idebenone; left eye: MD −4.37 dB, *p* < 0.5%—at diagnosis vs. −1.85 dB, *p* < 0.5%—after 3 months of Idebenone) (Figure 5) and a subjectively perceived improvement. Case number three presented CRR.

Although both patients received treatment for one year, they missed their scheduled follow-up appointments at 6 months and 1 year. We were able to arrange phone consultations instead. Case number two did not report any noticeable improvement after one year of treatment and opted to discontinue Idebenone administration. In contrast, patient number three reported sustained vision after the 3 month-follow-up. Unfortunately, after that period, the patient relocated abroad, ceased treatment, and became uncontactable for further follow-up.

**Figure 6:**
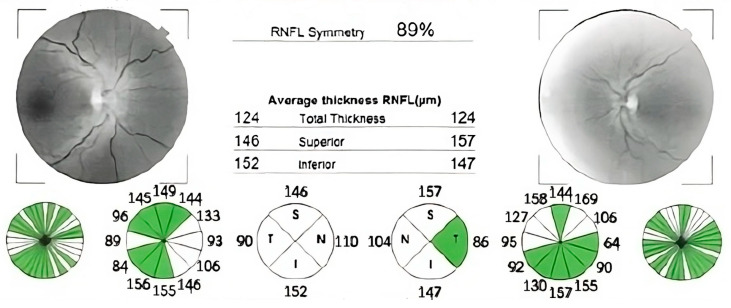
OCT reports showing bilateral retinal nerve fibers swelling.

Case number four was a 46-year-old man, referred to us for suspicion of subacute stage of LHON. The patient complained of subacute vision loss in both eyes, developed sequentially, over a period of approximately 3 weeks. Initially, his right eye was affected, followed by the left eye after two weeks. At the time of presentation, which was one month after the onset of symptoms, his visual acuity was 1.80 logMAR in both eyes. The intraocular pressure and the anterior segment exam were normal. Dilated fundus examination revealed pseudo-edematous optic discs, with peripapillary nerve fibers layer swelling, tortuous retinal vessels, and peripapillary telangiectasias. The OCT reports confirmed the appearance of the optic discs (Figure 6). Additionally, the 24-2 Humphrey VF test indicated ceco-central scotomas and enlarged blind spots.

Case number four was the only patient diagnosed in subacute stage of the disease. After genetic testing confirmed the diagnosis, we advised starting Idebenone therapy immediately, but, unfortunately, treatment was delayed by two months due to circumstances beyond our control. During this time, the patient’s VF defects worsened. In the course of follow-up visits, we evaluated the patient by means of visual field, OCT, and visual acuity. Following the start of treatment, the 24-2 Humphrey VF showed a decline for 17 months. However, we later observed fenestrations in previous scotomas, with noticeable improvement in VF for the left eye and a relatively stable MD for the right eye (Table 1).

**Figure 7:**
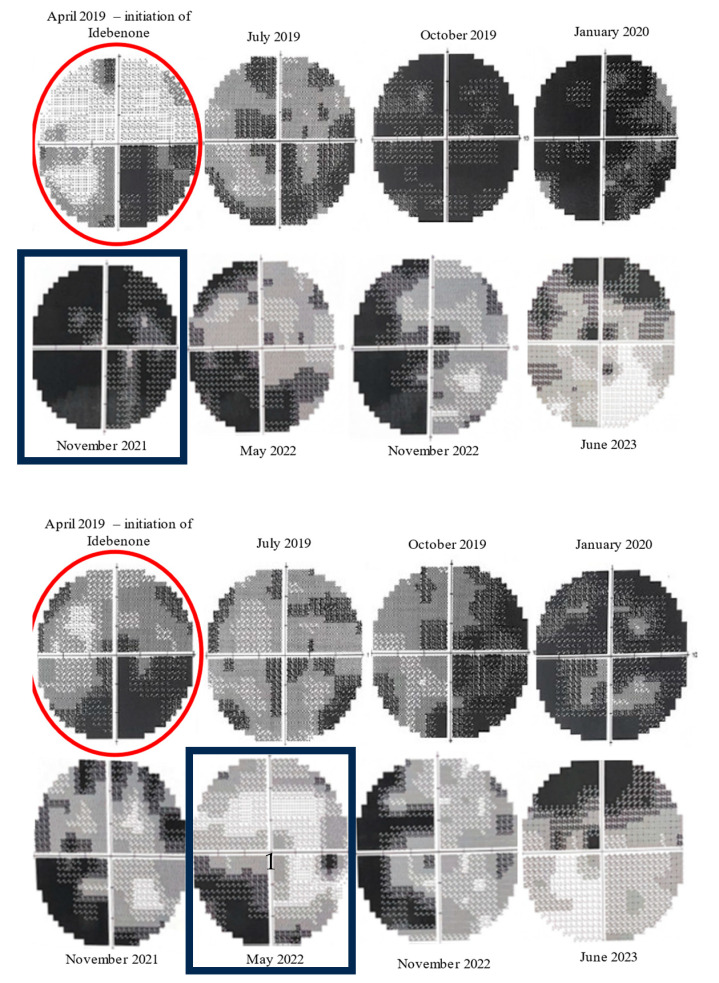
Evolution of 10-2 Humphrey VF from initiation of Idebenone (**top**: right eye, **bottom**: left eye). Initially, there was a decline in visual fields, but, after approximately 30 months of therapy (November 2021), there was a noticeable improvement in the central scotomas of the left eye. The improvement was noticed later (May 2022) in the right eye. (red circles—the moment of Idebenone initiation, black rectangles—the moment of improvement of visual fields).

**Figure 8:**
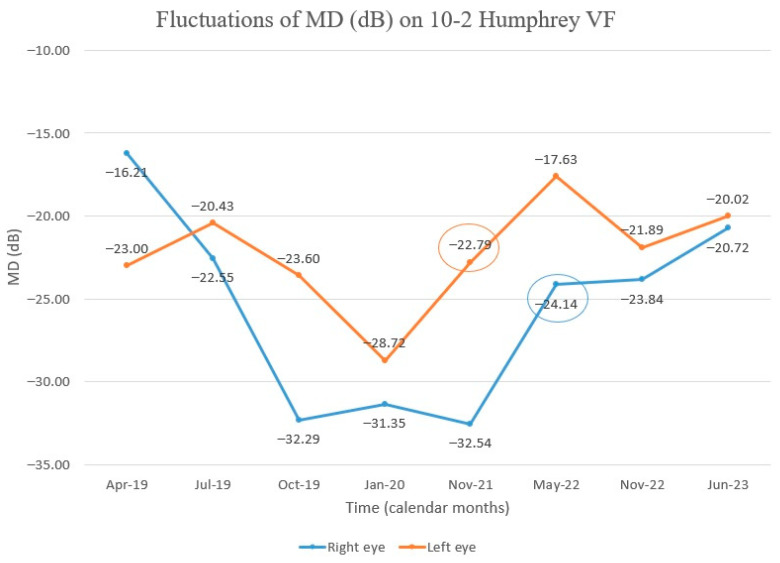
Chart showing MD fluctuation on 10–2 Humphrey VF after Idebenone therapy was started. Initially, there was a decline in MD, but we observed subsequent improvement, first in the left eye. (Encircled values—MD values at the time of improvement of visual fields after the initial decline).

Throughout the monitoring process, we utilized the 10-2 Humphrey VF as a means of gaining a better understanding of the central vision (Figure 7). We initially observed a decrease in the central visual field but later noticed an upward trend in the mean deviation (MD), beginning with the left eye 30 months after Idebenone initiation. Further evaluation demonstrated improvement in the right eye as well. The VF in the right eye continued to improve during our last assessment, but, in the left eye, the value of MD fluctuated negatively with a slight decrease, followed by an upward trend (Figure 8). Despite treatment being stopped three months prior to our last evaluation, the positive outcome was still maintained. Regrettably, the patient missed the scheduled visit on the last day of treatment.

The patient’s visual acuity remained stable and showed no improvement for one year and a half after starting Idebenone treatment. At the follow-up visit after 18 months of therapy, the patient was able to read five letters on the ETDRS chart from a distance of 1 m with both eyes (with +0.75 sphere addition), equivalent to 1.6 logMAR. The patient also subjectively noted the improvement in vision. The vision remained stable (1.6 logMAR) at the last visit (June 2023), despite stopping Idebenone 3 months prior. During treatment, the patient presented CRR and, therefore, was recommended to undergo a 36-month Idebenone regimen.

**Figure 9:**
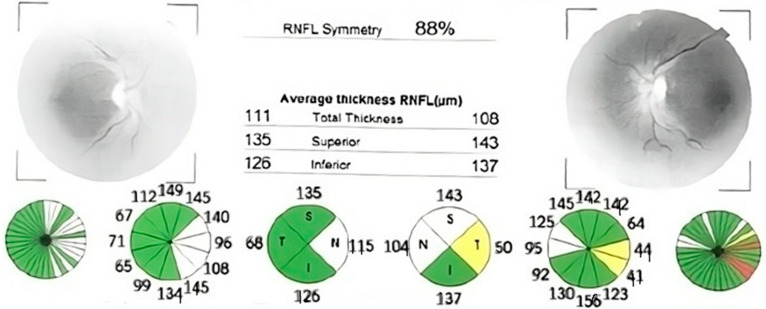
OCT report after 3 months of treatment, showing reduced RNFL edema compared to the moment of diagnosis. The left eye report shows the beginning of temporal atrophy.

**Figure 10:**
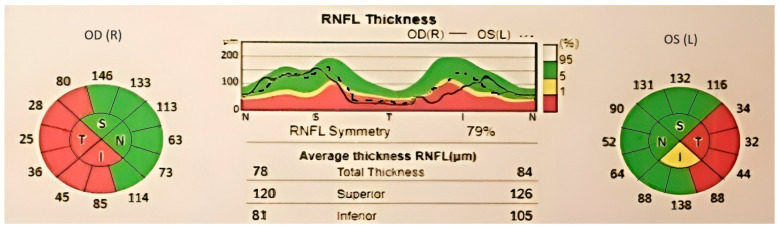
OCT report after 9 months of treatment. We can notice reduced RNFL thickness in temporal and inferior quadrants.

OCT scans showed a gradual decrease in the thickness of the retinal nerve fiber layers starting from edematous appearance at diagnosis (Figure 6), followed by a trend towards false normalization of thickness (Figure 9) and then atrophy in the temporal quadrant with sparing of fibers nasally (Figure 10). Gradually, the patient started to lose fixation, and it became harder and harder for technicians to obtain good OCT captures. The last reports obtained were unreliable and difficult to assess.

The treatment was taken for a total of 36 months, with interruptions due to delayed prescription acquisition, considering the patient lived in another town.

## 4. Discussion

The cases presented highlighted the importance of prompt and accurate diagnosis and treatment of LHON disease.

Case number one proves the natural history of disease, LHON leading to irreversible vision loss and significantly impacting a person’s quality of life. Therefore, finding the best treatment is crucial. Moreover, a differential diagnosis correctly constructed can save a lot of time and can facilitate the early start of treatment. LHON can be easily misdiagnosed as optic neuritis. MRI can assist in diagnosing optic neuritis [21,22], but imaging features can also resemble LHON. Other conditions that should be ruled out when diagnosing LHON include toxic optic neuropathy, vasculitides, infections, and compressive or infiltrative optic neuropathy.

Cases number two and three perfectly describe the familial aggregation of LHON cases. The ailment impacted males spanning three generations and one female in the most recent generation. The mother of our patients was a carrier, and her brother had the disease. Earlier in the family history, their grandmother was also a carrier of LHON, and her brother manifested the condition. Unfortunately, we do not know much more about the family’s pedigree. The pattern of LHON transmission is well known; therefore, genetic counseling should be provided for affected families, though incomplete penetrance provides multiple possibilities of disease manifestation. The condition has a non-Mendelian pattern of inheritance [4,23]; it is maternally inherited, so females who carry a mutation will pass it to all their children. Thus, all the siblings of an affected patient, his/her mother, and the mother’s siblings should be aware of the risk they have. Moreover, their lifestyles should be changed, if the case requires, in order to try to avoid developing or worsening the disease; tobacco and alcohol should be avoided. Case number two was advised to give up smoking in order to reduce oxidative stress, but he did not comply with the recommendation.

Both brother and sister received therapy, even though the disease had already started over a year before diagnosis. According to a study by Pemp et al. [24], patients who received treatment during both the early chronic phase (1–5 years after clinical onset) and the late chronic phase (>5 years) reported positive outcomes. Previous clinical studies mostly focused on patients in the acute phase of disease; therefore, the consensus [19] proposed that Idebenone should be initiated within the first year after onset. However, recent reports have shown that clinically relevant improvement is mostly seen after 2 or 3 years of treatment, when the disease has already progressed to the chronic stage [24,25]. This suggests that starting Idebenone treatment later after the onset of symptoms may be appropriate. It seems that retinal nerve fibers affected in optic neuropathies (even in anterior ischemic optic neuropathy) may be only injured, exhibiting dysfunctional behavior and can be reactivated after a period of inactivity [26]. Nevertheless, while Idebenone can be administered at a later time, prompt diagnosis is critical, as studies have shown that early treatment administration can result in a lower number of retinal nerve fibers being lost over time [17].

Unfortunately, case number two made the decision to cease their Idebenone therapy after one year of treatment due to perceived lack of improvement in visual acuity. It is important to note, however, that changes in clinical condition may become apparent later on in the treatment process. Therefore, it is recommended that patients do not prematurely discontinue their treatment, even in cases of chronic illness, simply due to a lack of early visual improvement.

We are sharing the results obtained with case number four, as we managed to continue the treatment for 36 months and we succeeded in evaluating the results and the impact of Idebenone administration. Although previous studies have shown the effectiveness and safety of Idebenone for patients with LHON, the duration of treatment is still a topic of debate. According to Catarino et al. [15], the probability and extent of recovery are positively correlated with the length of treatment. Therefore, they recommend continuing Idebenone for at least 18–24 months in order to improve the likelihood of achieving a CRR. Mashima et al. [27] supported the same idea, showing that the approximate time to recovery during treatment is 17 months. Patient number four achieved a CRR after 18 months of treatment, and Idebenone was continued for another 22 months, resulting in a stabilization of vision. However, it should be noted that the treatment was not administered continuously. Although Idebenone was prescribed every 3 months, the patient was unable to come for every prescription on time, resulting in deferred treatment. Despite this, during the last visit, which occurred 3 months after the cessation of Idebenone treatment, we observed that good outcomes in terms of visual acuity, and visual fields were still maintained. Our findings were consistent with other studies in medical literature on this topic. The RHODOS study [17] was followed by RHODOS-OFU (observational follow-up) [18], which demonstrated that the positive effects achieved after a 6-month Idebenone treatment were still present about 30 months after discontinuation. The authors suggested that the progression of the disease after treatment followed the same pattern as the natural history of LHON, with stabilization of vision after reaching a nadir, also observing a limitation of RGCs loss after this point. Using Idebenone during the acute or subacute phase, when RGCs become dysfunctional, helped to keep these cells viable, restoring their function, and maintaining it even after Idebenone was no longer administered.

Regarding the natural evolution of the disease, a study conducted by Liu et al. on a Chinese population with LHON (m.11778G>A) revealed that visual field defects progress rapidly within the first 6 months after disease onset but stabilizes after 9 months [28]. This underscores the significance of early treatment in reducing the number of retinal ganglion cells affected and prone to death. Case number four benefited from prompt treatment initiation, but still experienced visual field deterioration at the time of first Idebenone administration. However, the only female subject in our paper showed a positive response to therapy, despite its initiation 2 years after onset. The optimal timing and duration of treatment for LHON disease are still a topic of debate and case reports presenting different types of evidence are crucial.

Patients with LHON disease may have predictive factors that influence their evolution to some extent.

There are articles that indicate females may have poorer visual outcomes [29], but patient number three had good evolution after treatment with CRR. This favorable result may be explained by the patient’s young age and relatively good visual acuity when she began taking Idebenone. On the other hand, optic disc edema and hyperemic aspects of the disc are considered negative predictive factors, as they indicate axonal stasis and secondary anterior ischemic optic neuropathy [23,29,30,31]. Our patient, diagnosed in the subacute stage, presented with these features, signaling at the beginning of treatment a potential unfavorable response to therapy. Nevertheless, he had a good outcome, translated by CRR and improved VF. Another predictive factor was the type of mutation found. All of our patients had the 11778 G>A mutation, which was the most common mutation and was considered to offer the worst visual outcomes [32,33].

**Scheme 1:**
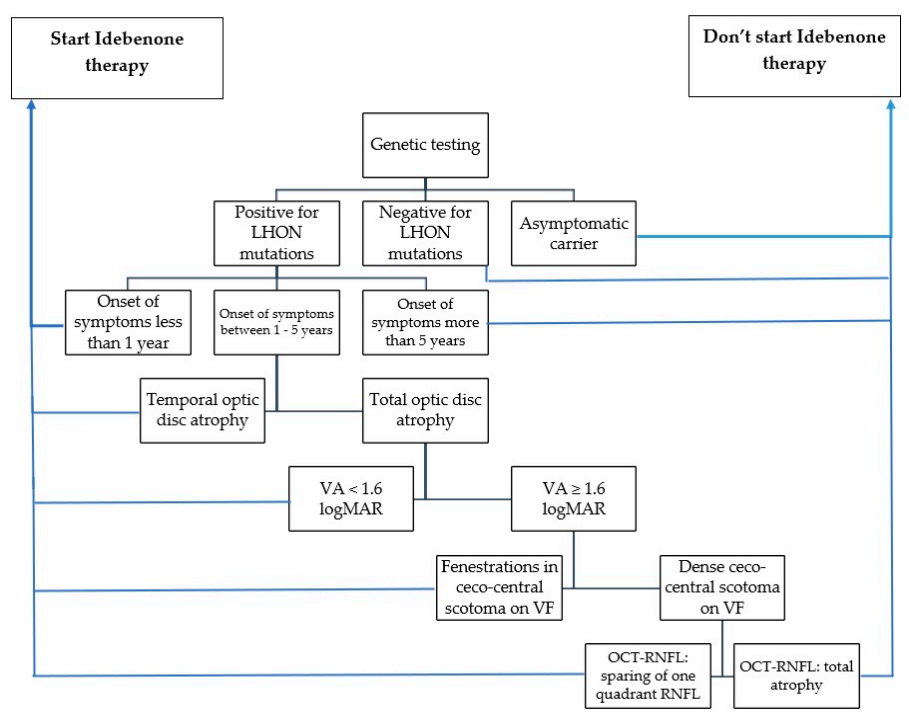
Suggested guideline for selecting the patients with indication for Idebenone administration (VA: visual acuity, VF: visual field, OCT: optical coherence tomography, RNFL: retinal nerve fiber layer).

After analyzing negative predictive factors and the evolution of our patients, we developed a guideline model (Scheme 1) for identifying patients who are likely to respond positively or negatively to Idebenone therapy. When considering whether to initiate Idebenone therapy, the following parameters should be taken into account: the timing of symptom onset, optic disc appearance, visual acuity, visual field appearance, and degree of optic nerve atrophy revealed by OCT-RNFL.

When evaluating the effectiveness of Idebenone administration, the main parameters to consider are visual acuity, visual field defects, and the appearance of the OCT-RNFL. In our study, only case number four showed different patterns of optic nerve structural changes during LHON evolution. According to a recent study [34], the stage of LHON can be determined by the thickness of the RNFL at a certain moment. A thickened or normal RNFL indicates subacute stages, whereas a thinned RNFL indicates longstanding disease. Monitoring these changes through OCT can help practitioners evaluate and correlate structural and functional aspects. Another study published in 2021 [24] showed that patients who received early treatment experienced gradual RNFL loss in the first year, followed by stabilization that was similar to our last patient’s evolution. Conversely, the same study found that patients with chronic disease had stable RNFL thickness on OCTs. Despite OCT RNFL showing the beginning of atrophic process in temporal quadrant after 9 months of treatment, our patient demonstrated functional improvement in visual acuity and visual field. As we mentioned earlier, it appears that dormant RGCs can be reactivated to regain function [26]. Similarly, a study of seven LHON patients with recent onset of symptoms [35] found that RNFL thinning did not correlate with final visual acuities and visual fields. The authors recommended the initiation of Idebenone treatment despite RNFL loss detected through OCT.

An important discussion we can debate is about the results demonstrated by case number four during the last evaluation. Visual acuity was stable, and central visual fields (10-2) showed further improvement. We suggest that our patients should benefit from a prolonged Idebenone regimen. Therefore, correlating our results with many others we pointed out in our paper, we recommend that Idebenone treatment should be continued for at least one year after stabilization in visual acuity and visual field, for a patient with CRR, regardless of the duration of treatment until visual stabilization. Even if one of these parameters registers earlier stabilization, Idebenone treatment should be continued one year after the other parameter stops improving too. However, a patient without positive outcomes during Idebenone therapy may continue the treatment for at least 36 months.

Our study deals with one of the rare diseases. Finding the right treatment for these conditions is crucial. Less than 5% of rare diseases have an effective treatment [36]. Moreover, a study that analyzed the diagnostic process of people with rare diseases from Spain found that patients visit approximately 10 healthcare professionals until finding the right diagnosis. They also wait over 6 months between first symptoms and first medical visit [37]. These diseases raise important concerns, so it is important to value any case and any experienced practitioners present.

Our descriptive study has some strengths: the long follow-up period, the large number of visits for evaluation, the different stages of diseases our patients have, and the presence of both males and females in our study group. However, our study has also some limitations. Our experience with LHON is from a single center, the study deals with a small number of patients, and all of them have the mutation 11778G>A in MT-ND4. However, large groups of patients are difficult to find for this rare disease.

## 5. Conclusions

Studying rare diseases is a difficult process. Clinical trials are usually constructed and conducted with important efforts, considering the low prevalence of these diseases in the general population. Therefore, isolated cases become major sources of information. Taking into account all these issues, we consider our work appropriate and relevant. The cases presented provide valuable data for management of patients with LHON. Our paper demonstrates the experience we gained with the management of LHON patients, mutation 11778G>A in MT-ND4, and has the role of completing previous studies on this subject. We presented the importance of correct and rapid diagnosis. However, we showed that Idebenone can also be effective when initiated during the chronic stage of disease. We emphasized the response to long-term regimens of Idebenone, pointing out the significance of extended period of treatment, in order to obtain a favorable outcome.

The complexity of LHON requires continuous research work, in order to find the best therapy needed for minimizing loss of vision and negative impacts on patients’ lives.

## Figures and Tables

**Table 1:** Evolution of MD (mean deviation) values on 24-2 Humphrey VF for patient number four.

Date	MD Values for the Right Eye	MD Values for the Left Eye
February 2019 (diagnosis)	−4.17 dB, *p* < 0.5%	−6.16 dB, *p* < 0.5%
April 2019 (initiation of Idebenone)	−13.26 dB, *p* < 0.5%	−20.76 dB, *p* < 0.5%
July 2019 (3 month-follow-up)	−19.52 dB, *p* < 0.5%	−22.34 dB, *p* < 0.5%
October 2019 (6-month-follow-up)	−26.39 dB, *p* < 0.5%	−27.21 dB, *p* < 0.5%
January 2020 (9-month-follow-up)	−26.68 dB, *p* < 0.5%	−28.47 dB, *p* < 0.5%
September 2020 (17-month-follow-up)	−26.75 dB, *p* < 0.5%	−17.61 dB, *p* < 0.5%

## Data Availability

Data analyzed in our article is contained within this published paper.
